# The College Controversy

**Published:** 1871-07

**Authors:** 


### "THE COLLEGE CONTROVERSY."

With this number the COMPANION closes the "medical controversy," so far as its editors are concerned. The questions involved in it are not local, but genera.. These we have attempted to discuss without animosity or "personal feeling," as questions of principles involving the ethics, independent of persons. We have discussed the law—not men. We do not desire to engender strife, or to prolong a controversy which threatens to degenerate into personalities. We will not become parties in such a contest. We appeal to the facts, and submit the case to the profession. We beg them to investigate these facts, independent of men or party feeling, upon their merits. In future, as in the past, this Journal will be devoted to the promotion of Science and the discussion of principles without reference to men or local issues.

